# Water Quality, Nutritional, Hematological, and Growth Profiles of *Ompok pabda* Fish Fry Reared in Biofloc Technology and Traditional Culture System with Different Stocking Densities

**DOI:** 10.3390/ani14010090

**Published:** 2023-12-27

**Authors:** Prianka Paul, Md. Sherazul Islam, Abul Farah Md. Hasanuzzaman

**Affiliations:** 1 Fisheries and Marine Resource Technology Discipline, Khulna University, Khulna 9208, Bangladesh; prianka@just.edu.bd; 2 Department of Fisheries and Marine Bioscience, Jashore University of Science and Technology, Jashore 7408, Bangladesh; dms.islam@just.edu.bd

**Keywords:** pabda, biofloc, weight gain, FCR, production

## Abstract

**Simple Summary:**

*Ompok pabda* fish has been increasing in market price because of its good taste and soft bony texture; thus, intensification of pabda cultivation by increasing stocking density can be an option to satisfy this market demand. However, intensification may result in some negative impacts on the culture environment and fishes, including water quality deterioration and production loss. Biofloc technology (BFT) is an environment-friendly approach for producing fish with higher stocking density. The present study aimed at assessing the effect of different stocking densities on the water, nutritional, hematological, and growth parameters of pabda fry reared in BFT and a traditional culture system (TS) in indoor tanks. The experiment had stocking densities for the treatments of 17 (TS_1_) and 22 (TS_2_), 17 (BFTS_1_), 22 (BFTS_2_), and 27 (BFTS_3_) fish/m^2^. Biofloc was developed in tanks using molasses as the carbon source (C:N = 20:1). Compared to the TS, the BFT system had a good index for water quality, nutrition, and growth of fish. Taking into account feed intake and benefit–cost ratio values, a stocking density of 22 fry/m^2^ was found optimal for the *O. pabda* BFT system. Globally, the data of this study may be useful for the aquaculture society to increase pabda and other catfish production in a sustainable way.

**Abstract:**

The present study evaluated water quality, immune responses, nutritional condition, and production of *Ompok pabda* fry (0.29–0.31 g) reared in a Biofloc technology (BFT) system (C:N = 20:1; molasses as organic carbon source), compared to the traditional culture system (TS; farmer’s practice). The experiment had stocking densities for the treatments of 17 (TS_1_) and 22 (TS_2_), 17 (BFTS_1_), 22 (BFTS_2_), and 27 (BFTS_3_) fish/m^2^. The fishes were fed at 3–10% of their body weight, and reared in cemented tanks for 90 days. Regarding water quality, dissolved oxygen (DO), pH, and total ammonia nitrogen (TAN) levels varied significantly (*p* < 0.05) between the traditional and BFT tanks. The highest specific growth rate (SGR) was in the BFTS_1_-reared fishes (4.11 ± 0.17) but the lowest was in the TS_2_-fish (3.51 ± 0.05). The fish reared in BFT had higher levels of protein, lipids, polyunsaturated fatty acids, essential amino acids, hematocrit, and neutrophil than the fish reared in TS tanks. Moreover, 98.33% survival was recorded in the BFTS_1_ while 86.67% was in the TS_2_. The highest BCR was estimated for the BFTS_2_ (1.22). Taking into account FCR and BCR values, a stocking density of 22 fry/m^2^ is likely practicable for an *O. pabda* BFT system.

## 1. Introduction

The pabda catfish (*O. pabda*) is a native potamodromous species that is classified in the family Siluridae within the order Siluriformes. It has a widespread distribution geographically across Bangladesh, India, Pakistan, Burma, and Afghanistan [1]. Pabda can be found in various wetland environments throughout Bangladesh, including flooded fields [2] and rivers, ponds, streams, and pools [3]. However, because of significant modifications in the ecosystems of the inland water bodies in Bangladesh, this small catfish is now considered endangered [4].

Pabda has a high market price because of its exquisite flavor, soft bony texture, and abundant lipoprotein content, which have made it popular among people in Bangladesh, East India, and Northeast India [5]. Due to consumer demand, this catfish is becoming a promising aquaculture species in India and Bangladesh. With a total fish production of 4.62 million metric tons (MT) in 2020–2021, Bangladesh is one of the top fish-producing countries in the world as a whole, and catfish production accounts for 73,180 MT of that total [6]. In Bangladesh, catfish farming is a part of aquaculture. The success in captive breeding as well as production of *O. pabda* fry in the hatchery has now made the hatchery-produced *O. pabda* fry accessible to farmers; both large-scale and small-scale farmers are progressively cultivating this little catfish species. The *O. pabda* is mostly cultivated in the districts of Mymenshing, Jashore, Khulna, Pabna, and Dinajpur in Bangladesh. Several approaches have been developed for cultivating Pabda, including semi-intensive management [7], earthen ponds [8], and a cemented canal [9]. The cultivation of pabda in ponds has recently benefited from the utilization of bottom-clean raceway technology [10].

In relation to the market demand for pabda, its farming with greater stocking density, feed supplementation, and health management drug use is likely to be intensified. However, it has been asserted that such intensification in aquaculture technologies will have an impact on the ecosystem of rivers, lakes, oceans, and other aquatic bodies, as well as the diversity of fish and other aquatic animals. Major issues of the aquaculture endeavors include husbandry practices, farm effluent release, excessive reliance on fishmeal-based feed, and disease breakout [11]. The primary excretory products of aquatic animals that build up in the aquaculture system are inorganic nitrogen species (ammonium NH_4_^+^, nitrite NO_2_^−^) [11]. Fish can only keep 20–25% of the protein of the feed they are fed, and the rest is excreted as ammonia and organic N in their waste and leftover feed in intensive conditions [12]. Moreover, use of chemicals, like antibiotics, for preventative purposes can have negative effects on wildlife and the environment. To reduce these negative consequences and preserve the environment’s sound health, improved and/or novel aquaculture technologies have been sought after. Recirculation aquaculture systems (RASs) and Biofloc technology (BFT) culture systems are two modern, cutting-edge aquaculture techniques that are widely used. However, an RAS is unable to recycle feed leftovers, likely leading to the NO_3_^−^ level increase in the aquatic environment and is somewhat costly. Accordingly, excellent water quality and enhanced fish biomass output may be achieved with relatively minimal investment and low operational expenses using BFT, which is an excellent revolutionary technique [13]. A BFT production system allows for the management of water quality with little to no water exchange in addition to creating nutrient microorganism-based feeds for the fish within the system [14]. External organic carbon is used to support the formation of a heterotrophic population that immobilizes ammonia [15] and make the system functional.

Biofloc Technology (BFT) is an organic, environmentally friendly, and cost-effective system for aquaculture ventures [16]. BFT is frequently employed in finfish culture [17] as well as shrimp farming [18]. Since the early 1990s, multiple studies have evaluated the potentiality of BFT in grow-out farming and seed production of commercial species such as catfish *Clarias gariepinus* [19,20], *Ictalurus punctatus* [21], and *Oreochromis niloticus* [22,23,24,25,26]. These studies have demonstrated the role of BFT in water quality improvement, growth, reproduction, immune system modulation, feed efficiency, and production. The effective manipulation of the C:N ratio through or in addition to the BFT system enhances biofloc growth; the heterotrophic bacterial load is balanced and ammonia concentration is controlled in the culture units [17,27]. In addition to being an effective way to reduce ammonium and nitrite buildup in culture systems, bioflocs developed in the water column of the culture can also provide in situ nutrients like protein, lipid, amino acids, and fatty acids [18,24,28,29]. Compared to fish cultivated in clear water (without preparation for biofloc development), fish (*Oreochromis niloticus*) raised in the BFT system have been found to grow more quickly [23,30,31].

It is also crucial to standardize the ideal stocking density in an intensive culture system like BFT to achieve appropriate goals for production. Stocking density has a considerable impact on fish growth and development as well as the stress of transporting fish in aquaculture water. Therefore, establishing the optimal density is essential to ensure the economically viable fish production [32]. It has been demonstrated that different stocking levels have both positive and negative effects on the growth of several fish species [33,34,35]. As a result of under-utilizing the already existing resources, below-optimal densities could result in poor growth and decreased overall production [36]. In general, overstocking can lead to a decline in water quality, physiological problems, and finally the undergrowth of aquatic organisms [37,38]. Investigating the stocking density appropriate for the growth, physiological well-being, digestion of digestive enzymes, improved production, and effectiveness of farmed fish is therefore a fundamental issue. Moreover, if none of the aforementioned factors change, increasing stocking density can result in higher yields.

Even though a wide variety of fish species have been produced using the BFT system, Bangladesh is still in the process of developing this technology [39,40]. BFT can be considered a good method for pabda production with rising stocking density because of the fish’s high monetary value, high market demand, and widespread availability; De Schryver et al. [41] has reported better growth, greater yield, and good revenues from *O. pabda* culture systems. Nevertheless, the farmers practice traditional farming system methods with an average stocking density of 17–22 fry/m^2^. To the best of our knowledge, no thorough studies of *O. pabda* cultivation in a BFT system have been conducted to date in Bangladesh; hence, the ideal stocking density in a BFT system for *O. pabda* farming under the environmental conditions of Bangladesh is still unknown. Stocking density is a decisive component in determining the efficiency and profitability of industrial-scale operations of fish species [42]. Therefore, the purpose of this study was to compare the efficiency of *O. pabda* in a BFT system to that of the traditional culture system currently utilized by farmers in Bangladesh in order to identify the effect of stocking density on water quality and production performance.

## 2. Materials and Methods

### 2.1. Experimental Design

**Figure 1:**
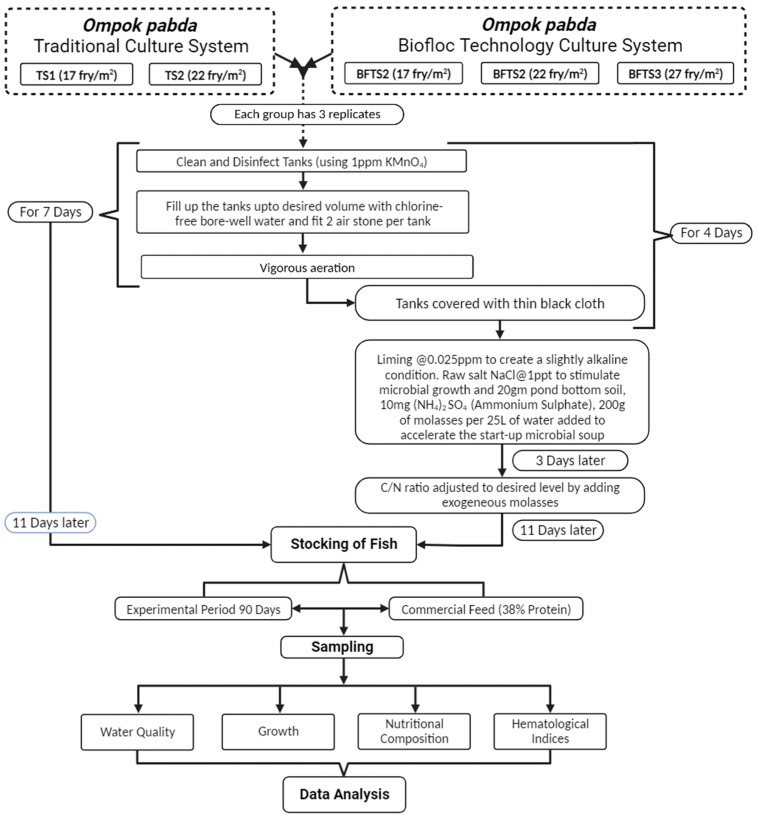
The framework to illustrate the analysis procedure of the present study.

The current study spanned a total of 90 days from March to June 2022, all of which took place in the wet lab of the Department of FMB (Fisheries and Marine Bioscience) at Jashore University of Science and Technology in Jashore, Bangladesh. Figure 1 illustrates the indoor experimental set up, which consisted of 15 (1.85 m × 1.22 m × 0.70 m) cemented tanks (1500 L) with 750 L water in each tank. Each tank had a blower installed (0.08 horsepower) and was connected to a PVC pipe inside to provide aeration, and each had two air stones for vigorous agitation. It is necessary to mention that no temperature control was used and no water exchange was performed throughout the culture period. The indoor environment was kept at a natural photoperiod of 12 h.

The current study was conducted using five treatments each with three replications: the stocking density of traditional culture system (i.e., the cultivation system being currently practiced by the local pabda fish farmers) were 17 fry/m^2^ (TS_1_) and 22 fry/m^2^ (TS_2_) and for the BFT, three individual treatments of BFTS_1_, BFTS_2_, and BFTS_3_ were established with 17, 22, and 27 fry/m^2^, respectively. The range of the stocking density 17 and 22 fry/m^2^ was obtained from the baseline survey among the pabda farmers of the Jashore region and the stocking density of BFTS_3_ was assumed to assess the higher stocking density in BFT. The traditional culture system involves culture of fish in tanks or artificial enclosures without addition of organic carbon source. According to the methodology outlined by Debbarma et al. [43], water from a bore well, devoid of chlorine, was used to fill all the experimental tanks, and the tank water was intensely ventilated for four days prior to the start of the research. Before stocking fry, a KMnO_4_ solution of 5 ppm was used to sterilize all of the experimental tanks. The tanks were covered with a thin black cloth to generate the diffused lighting environment necessary for the quick growth of the heterotrophic populations. Molasses (24% *w*/*w* carbon) was chosen since it is one of the most regularly used or investigated ingredients in biofloc production [43,44,45], easily accessible nearby, and a low-cost product. In accordance with the theory of Avnimelech [17], the previously weighed organic carbon source was mixed with the feed containing 38% crude protein daily to maintain a C:N ratio of 20:1 for encouraging biofloc growth in the tanks. Throughout the experiment, no water in any treatment was exchanged.

### 2.2. Fish Stocking

Twenty-five-day old *O. pabda* fry (weight range 0.29–0.31 g and length range 3.33–3.43 cm) were bought locally and then transported in a sealed plastic bag (0.5 m^3^) containing 1 kg fish and oxygen-saturated water. The fish were acclimated for 48 h in rectangular cement tanks (1500 L) without food for 24 h. Following a period of acclimatization, the quality of the fish was evaluated based on their morphological characteristics and physical activity. According to the experimental design, 40 fry in each TS_1_ tank and 50 fry in each TS_2_ tank of the traditional system were stocked; in the cases of the BFT treatment groups, 40 fry/tank for BFTS_1_, 50 for BFTS_2_, and 60 for BFTS_3_ were stocked. Fish fry were stocked randomly in all tanks, and were reared in the experimental conditions for 90 days.

### 2.3. Feeding Management

The fish were fed a 38% protein-containing commercial diet (pellet feed; Nourish Fish Feed Co., Ltd., Dhaka, Bangladesh) during the experiment. As per the farmer’s practice extracted from the baseline survey, the feeding frequency ‘twice a day’ (7:00 and 19:00) was assumed and maintained. The feeding rate ranged between 3 and 10% of the total fish biomass; for the first 15 days, feeding was determined at 10% of fish biomass and then it was gradually adjusted to 3% considering their feeding behavior and weekly weight gain. The necessary amount of molasses containing 24% carbon was calculated based on the 38% protein level of feed and was added after each feeding in the BFT tanks [43].

### 2.4. Floc Formation and C:N Ratio Management

A C:N ratio of 20:1 was maintained in the BFT tanks by using molasses (C_6_H_12_NNaO_3_S) (24% *w*/*w* carbon), which was estimated following calculations by Avnimelech [17] and Debbarma et al. [43] with some minor modifications. The carbon content of molasses (C_6_H_12_NNaO_3_S) was assessed after purchasing it from a nearby market in Jashore, Bangladesh. The feed was administered taking into account the nitrogen and carbon contents of the feed as well as the carbon content of molasses. There was no carbon addition in the control group (traditional culture system; TS). Following the standard technique reported by Avnimelech [17], with suitable adjustments and the methodology detailed below, floc formation in the system was initiated 18 days prior to fish stocking.

After a thorough cleaning with 1 ppm KMnO_4_, the experimental tanks were filled with water. Once the desired volume (750 L) was reached, the tanks were outfitted with air stones/diffusers (2 nos. per tank). A mild alkaline state was achieved with liming at 0.025 ppm. In order to promote microbial growth for efficient floc formation, 1 ppt of raw salt (NaCl) was applied to each tank. To speed up the start-up of the microbial soup, an additional 10 mg of (NH_4_)_2_SO_4_ (ammonium sulfate), 20 g of bottom pond soil, and 200 mg of molasses were added. As shown in the equation ΔCH = feed × %N in feed × % N excretion/0.05 [17], after three days the C:N ratio was corrected to 20:1 by introducing molasses at a calculated value of 0.635 g for every one gram (1 g) of nourishment provided.

The determination of the required amount of organic carbon source (molasses) for the experiment was made two weeks after the fish were stocked. This determination was based on the assumption that 70% of the feed would permeate into the system. All tanks operated without any water exchange during the experiment, with the exception of the losses due to evaporation during sludge removal (5–10%). Preparations were made to add sodium bicarbonate (NaHCO_3_) to the tank to keep the pH of the culture water above the desired level (7.0). The sludge at the bottom of the culture tanks was emptied once a week.

### 2.5. Water Quality Assessment

All through the experimental period, water parameters were recorded. Daily measurements of pH, temperature, and dissolved oxygen (DO) were made using portable meters (respectively, Adwa AD-101, Smart sensor AR867, and Lutron PDO-519). The ammonia test kit (Hanna HI715) was used to assess the total ammonia nitrogen (TAN) level. Total dissolved solids (TDS) values were recorded every day in individual tanks with a measurement tool (HI-98302, Hanna). The other parameters, including salinity (EZ-9909-SP), alkalinity (Hanna HI3811), NO_3_ (nitrate; Hanna HI-3874-0), and NO_2_ (nitrite; Hanna HI-3873-0), were determined on a weekly basis. Following the methodology used by APHA et al. [46], biological oxygen demand (BOD) was assessed twice a week. An Imhoff cone was used to collect a water sample of 1000 mL, which was allowed to settle for 20 min to determine the floc volume, following the procedure given by Avnimelech [47].

### 2.6. Determination of Proximate Composition of Biofloc and Fish Carcass

Floc was collected after harvesting from each BFT tank using an Imhoff cone and filtered through a 100 μ mesh [48]. At the end of the experiment, ten fish were collected from each replication for the nutritional composition study, the test for moisture content was conducted for immediate analysis of the recently captured fish, and the entire fish was crushed and kept at −20 °C for subsequent analyses. According to AOAC [49], the contents of moisture, crude protein, lipid, and ash in the floc and fish carcass samples were calculated. The micro Kjeldahl method was used to measure crude protein, and the amount was then calculated by multiplying by 6.25. A soxhlet fat extraction device was used for extracting 2 to 5 g of the sample in a methanol and chloroform (1:2) solution; then, the crude lipid was measured from the fat extracted. In order to determine the ash content, the sample was burned in a muffle furnace for 24 h at 550 °C. Through oven-drying at 105 °C for 24 h, moisture was determined. The intermediate filtration method [50] was used to quantify the crude fiber content. After completing the analysis for ash, crude fiber, crude fat, and crude protein, the difference was used to estimate the nitrogen-free extract [51]. To determine amino acid profiles, a High Speed Bench Top Amino Acid Analyzer (LA8080) was used. For the fatty acid profile, the extraction of total lipid was carried out with a mixture of chloroform (20 mL) and methanol (10 mL) according to a modified Folch method and followed by fatty acid methyl ester (FAME) preparation and analyzed in Gas Chromatography-Mass Spectrometry (GC-MS) [52]. 

### 2.7. Growth Performance

Weekly measurements of the length and weight of ten (10) fish from each tank were taken and returned to their respective tanks after measuring the parameters. No sedative was applied during the operation. After 90 days of culture, the tanks were drained and survival was determined. According to dos Santos et al. [53], final body weight (FBW), final length (FL), weight gain (WG), specific growth rate (SGR), feed conversion ratio (FCR), survival rate (SR), and final biomass were estimated using the following formulas:

- Weight gain (g) = W_f_ − W_0_, where W_f_ = final weight and W_0_ = initial weight;
- Specific growth rate (%/day) = $\frac{100(InW_{t}-InW0)}{T}$ where, W_t_ = final weight (g) and W_0_ = initial weight (g) and T = rearing period (days);
- Feed conversion ratio = $\frac{\mathrm{Feed}\;\mathrm{intake}}{\mathrm{Weight}\;\mathrm{gain}\;(\mathrm{wet})}$
- Survival rate (%) = $\frac{100\left(\mathrm{Final}\;\mathrm{fish}\;\mathrm{count}\right)}{\mathrm{Initial}\;\mathrm{fish}\;\mathrm{count}}$
- Final biomass (kg/m^2^) = $\frac{\mathrm{Final}\;\mathrm{biomass}\;\mathrm{in}\;\mathrm{tank}}{\mathrm{Tank}\;\mathrm{volume}}$, where Final biomass = Final weight × Number of fish survived.

### 2.8. Hematological Measurements

Fifty fish were collected at random from each tank and anesthetized with 200 mg/L of clove powder [54] after 90 days of the experiment. The caudal peduncle was used to properly extract blood samples using a 1 mL hypodermal medical syringe (24 gauge needles; Jimi Syringes and Medical Devices Ltd., Dhaka, Bangladesh). To prevent hemolysis of blood cells, blood samples were immediately combined and transferred to a test tube that was thinly coated with the anticoagulant EDTA powder. Blood was drawn into Eppendorf tubes (no EDTA) for plasma collection, allowed to clot for 2 h, and then centrifuged for 15 min at 3000 rpm [55]. After that, the supernatant plasma was gathered and kept at −20 °C until hematological evaluations [54]. The KT-6390 (3 Diff) hematology analyzer (Genrui Biotech, Shenzhen, China) was used to estimate red blood cell (RBC), white blood cell (WBC), hematocrit (Ht)%, hemoglobin (Hb)%, and differential hematocyte counts (DHC).The levels of glucose and cholesterol in the blood were assessed with a biochemistry analyzer (ChemRead 3000).

### 2.9. Economic Evaluation

The benefit–cost ratio (BCR) was used to calculate the profitability of *O. pabda* production. Throughout the experiments, feed ingredients and *O. pabda* fry were purchased at the wholesale market price. Both fixed costs (FCs) and variable costs (VCs) were included in the total investment cost (TC) values. Fresh fish of varying sizes were used to calculate total revenue (TR; value of total production) based on the selling price at the local market.

The following metrics were evaluated using the following formulas:

BCR = TR/TC where TR = Total Revenue and TC = Total Cost

### 2.10. Statistical Analysis

Variations in water quality values were calculated using multivariate analysis of variance (MANOVA) and descriptive statistics (95% confidence level). Their values were reported as mean ± standard deviation. A two-way analysis of variance (ANOVA) was carried out, followed by a Tukey test, to ascertain the significance effect of the rearing system and stocking density on the production performance factors. Hematological index differences attributable to different culture systems were calculated using a one-way analysis of variance. In each case, the statistical threshold of *p* < 0.05 was applied to identify significant differences. All statistical analyses were performed using IBM SPSS Statistics 20, and Microsoft Excel 2016 was used to create the visualizations.

## 3. Results

### 3.1. Water Quality

In this investigation, there were differences in water’s physico-chemical parameters between the BFT and traditional culture systems (Table 1), but no such differences were detected between the treatments of the traditional system. Temperature (18.29–29.56 °C) did not differ significantly (*p* > 0.05) between the rearing systems, although there was significant weekly variation. In comparison with traditional culture tanks, the average levels of pH, DO, TAN, NO_2_, NO_3_, BOD, and alkalinity in the BFT tanks were significantly different. The variation in TDS values within the BFT treatments and between the two rearing systems was significant (*p* < 0.05). But hardness values did not show any significant difference between the two rearing systems. Throughout the experimental period, the floc volume (FV) of the BFT group increased until it reached a maximum volume of 14.57 ± 0.2 mL L^−1^ in BFTS_1_ treatment. The average FVs in the BFT tanks with low stocking density and high stocking density were 14.57 ± 0.2 and 13.97 ± 0.35 mL L^−1^, respectively.

### 3.2. Proximate Composition of Biofloc and Fish Carcass

**Figure 2:**
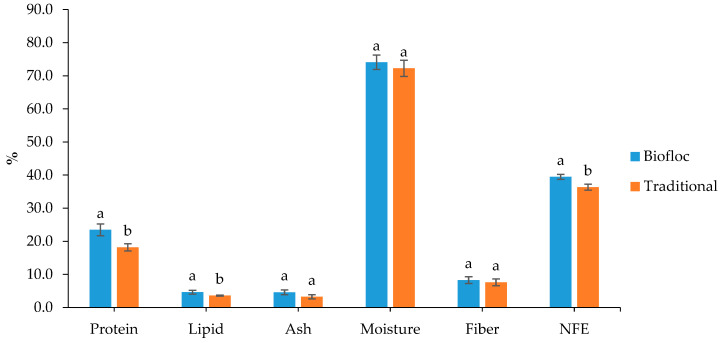
Proximate composition of the fish carcass (% dry weight); different letters indicate significant statistical differences (*p* < 0.05).

**Figure 3:**
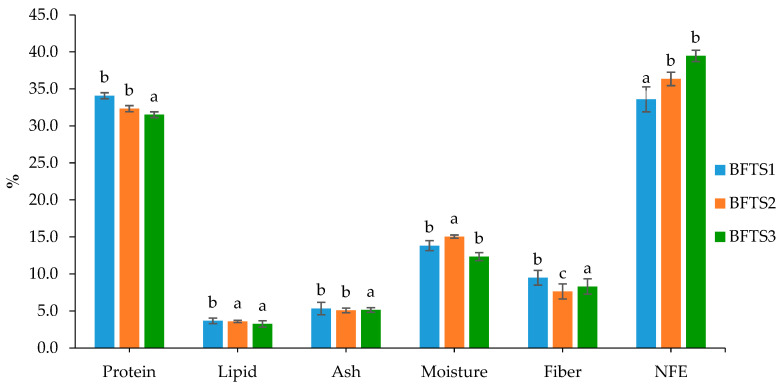
Proximate composition of the floc (% dry weight); different letters indicate significant statistical differences (*p* < 0.05).

Between the BFT and traditional systems, the *O. pabda* carcass’s proximate composition (% dry weight) significantly (*p* < 0.05) differed for protein (18.19–23.47%) and lipid (3.61–4.64%) content (Figure 2). However, the ash and moisture content did not differ significantly (*p* > 0.05) between the BFT and traditional groups. With different stocking densities, the nutritional composition of the biofloc varied in the BFT system (Figure 3); the BFTS_1_ had higher levels of crude protein (34.08 ± 0.41%), crude lipid (3.66 ± 0.38%), and ash (5.33 ± 0.83%). On the other hand, the content of NFE (nitrogen free extract) was higher in the BFTS_3_ (39.46 ± 0.77%) than in other treatments.

### 3.3. Fatty Acid Profile of O. pabda

Different fatty acid profiles were found in the fishes reared in the two culture systems; the fish reared in the BFT treatments had significantly (*p* < 0.05) higher levels of saturated fatty acids (SFA; 39.63 ± 0.74), monounsaturated fatty acids (MUFA; 28.18 ± 1.25), and polyunsaturated fatty acids (PUFA; 36.07 ± 1.41) than the fish of the traditional system (Table 2). However, there were no significant differences in linoleic and γ-linolenic acid content of traditional and BFT-reared fishes. The fishes from the TS tanks had a higher eicosapentaenoic acid (EPA) level while the BFT-reared fish exhibited significantly higher (*p* < 0.05) levels of α-linolenic acid, arachidonic acid, and docosahexaenoic acid. The BFT-reared *O. pabda* had a significantly higher muscle n − 3/n − 6 ratio (1.2) than had the TS-reared fishes (1.1).

### 3.4. O. pabda Amino Acid Profile

Essential amino acids (EAAs), conditionally essential amino acids (CEAAs), and non-essential amino acids (NEAAs) differed between the biofloc (BFT) and traditional (TS) rearing systems of *O. pabda* (Table 3). The BFT-reared fishes had significantly (*p* < 0.05) higher total EAA levels (74.6%) than the fishes reared in the traditional system (60.2%); among the EAAs, significantly higher levels of valine (16.1%), methionine (7.6%), lysine (5.2%), and leucine (5.1%) were also recorded in the fishes of the BFT system. The CEAA level of the BFT-reared fishes was also significantly higher. In contrast, a significantly greater level of total NEAAs was found in the fishes of the traditional tanks (61.9%), but BFT-reared fishes had significantly higher tyrosine and lower alanine contents.

### 3.5. Production Performance

The production parameters estimated as shown in Table 4 point out the influence of stocking density and rearing systems of *O. pabda*. Significant differences (*p* < 0.05) were found between the BFT system and the traditional system for FL, FW, WG, SGR, FCR, and survival rate. The growth metrics were significantly affected by the stocking densities. After 90 days, the mean final weights for TS_1_, TS_2_, BFTS_1_, BFTS_2_, and BFTS_3_ was 7.99 ± 0.17, 7.15 ± 0.58, 12.31 ± 93, 10.04 ± 0.14, and 9.98 ± 0.94 g, respectively.

**Figure 4:**
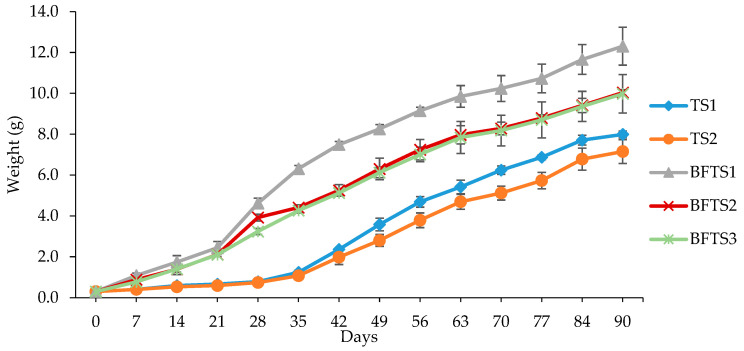
The growth performance (average body weight) of *O. pabda* reared in the biofloc (BFT) and traditional (TS) culture systems at different stocking densities.

Significant differences (*p* < 0.05) were observed between the treatments, with BFTS_1_ recording the highest final weight and TS_2_ the lowest (Figure 4). In the BFTS_1_ (4.11 ± 0.17), the SGR was found to be the highest, and in TS_2_ (3.51 ± 0.05), the lowest. The WG in BFTS_1_ was the highest (12.01 ± 0.92 g), followed by BFTS_2_ (9.73 ± 0.13 g) and BFTS_3_ (9.68 ± 0.05 g), and the lowest was in TS_2_ (6.85 ± 0.56 g). However, TS_2_ had the highest estimated FCR (1.73 ± 0.12), while BFTS_2_ had the lowest (1.27 ± 0.04). BFTS_1_ had the best survival rate (98.33 ± 2.89%), while TS_2_ had the lowest (86.67 ± 5.03%). The results of this investigation showed that the culture system had an impact on the production of *O. pabda*; BFTS_3_ (0.231 ± 0.02 kg) and TS_1_ (0.127 ± 0.01 kg) significantly (*p* < 0.05) obtained the highest and lowest production, respectively.

### 3.6. Hematological Parameters

**Figure 5:**
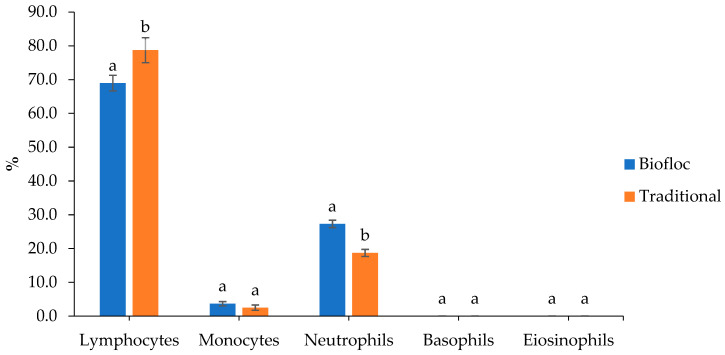
Differential hematocyte count (%) of *O. pabda* reared in biofloc (BFT) and traditional system (TS) at the end of the 90-day experimental period; different letters indicate significant statistical differences (*p* < 0.05).

This study unambiguously demonstrates that culture systems have an impact on the hematological profile of *O. pabda* (Table 5). Red blood cell counts (RBCs), hemoglobin (Hb), lymphocytes, neutrophils, and glucose of the fishes were all significantly (*p* < 0.05) influenced by the culture systems (Table 5; Figure 5). The fishes reared in the BFT system had higher RBC (3.28 ± 0.28), Hb (5.04 ± 0.26), hematocrit (34.50 ± 0.79), and neutrophil (27.3 ± 1.09) counts, but their glucose levels (9.06 ± 1.32) and lymphocyte (68.98 ± 2.31) counts were lower than those of fishes reared in the traditional system. The content of cholesterol (114.90 ± 5.36) and monocytes (3.66 ± 0.64) in the BFT system-reared fish were found to be higher but they did not vary significantly (*p* > 0.05) for the two rearing systems.

### 3.7. Economic Evaluation

The cost-effectiveness analysis reveals that the traditional system has the lowest estimated total cost, while the BFT system has the highest estimated revenue (Table 6). In terms of profitability, the BFTS_2_ treatment had the highest BCR (1.22), while the TS_1_ treatment had the lowest BCR.

## 4. Discussion

### 4.1. Water Quality

The maintenance of water quality is a crucial element in a biofloc technology for fish cultivation systems. There was no statistically significant difference in temperature between treatments (*p* > 0.05), and all water parameters were within the optimal range for the species [7].

In the current investigation, the DO level was kept above the acceptance level >5.0 mgL^−1^ [9] for the fishes by installing aeration systems in all the treatment tanks; the DO level was within the acceptable range for fishes [56]. There may be a correlation between the high stocking density in BFTS_3_ and the low dissolved oxygen level. In addition, the DO likely dropped in the BFT group because of the combination of increased respiration from animals and heterotrophic bacterial growth in the first phase, as is supported by Zaki et al. [57] and Wu et al. [58]. Throughout the experiment, the pH values of the TS were found to be alkaline. However, the pH levels measured in this investigation were within the ranges documented in the previous studies [9]. The BOD concentrations in the BFT treatments were greater than in the traditional system, which was caused by the growth of bioflocs because bioflocs have an oxygen requirement in the water [59]. However, as revealed by earlier research [60,61], the ranges of BOD values determined in this study were found to be satisfactory for fish production.

According to Avnimelech [47], FV (floc volume) is regarded as the primary indicator of biofloc generation. The floc formed, fish-excreta, other residues, and metabolic activity of the microorganisms in the in the BFT tanks’ water had influence on the alkalinity and hardness in the BFT system [62]. The amount of TAN, NO_2_, and NO_3_ varied significantly between the different rearing systems in the current investigation, with the BFT treatments recording the lowest levels. Notably, the nitrogen derivative levels remained under acceptable limits for aquaculture systems [63]. The rise in TAN, NO_2_, and NO_3_ levels was likely linked to higher stocking densities in both aquaculture systems. As pointed out by Minabi et al. [64] and Khoa et al. [65], there is an association of higher stocking density, abundance, and diversity of biofloc (e.g., heterotrophic and nitrifying bacteria). Though the level of nitrogenous compounds is affected by stocking density and biofloc availability [53], the current investigation did not discover any appreciable effects of stocking density on the buildup of nitrogenous derivatives. As TAN, NO2, and NO3 are acknowledged as noteworthy contributors to water pollution, this study demonstrated that the BFT system efficiently managed these parameters.

### 4.2. Production Performance

BFT has evolved as an environmentally friendly approach for improving fish productivity and maintaining water quality [23]. In light of this study, the BFT used in the current investigation offered more growth, survival, production, and a lower FCR than the traditional cultivation technique. It is of note that this study has also determined the effect of stocking density on production parameters, though the stocking density of 27 fry/m^2^ was not included as a treatment of the traditional farming system in which famers do not practice stocking densities greater than 22 fry/m^2^. Accordingly, this research work with non-orthogonal design did not report the effect of increasing stocking density (i.e., 27 fry/m^2^) in the traditional system but focused the performance of BFT system with higher stocking density. The outcomes were consistent with earlier findings; the BFT has resulted in better survival and production [53,66], but a lower feed conversion ratio for fish [53], in contrast to other form of farming systems like clear water, or recirculating aquaculture systems. The better production of BFT treatments in our study might be associated with forms of nutrition, biofloc’s dietary benefits, and better water quality, as also evidenced by other publications [23,66]. Therefore, the present study’s findings suggested that *O. pabda* might adjust to a BFT cultivation system.

The current study found that the BFTS_1_ tank had higher weight growth, SGR, and survival than the BFTS_2_ and BFTS_3_ tanks, which is similar to what previous studies reported as the impact of stocking density on fish production [53,67]. Tambaqui fish and GIFT tilapia weight gain decreased when density increased in a BFT system. Additionally, the stocking density exhibits this fluctuation in FCR, which may be correlated with the effects of microbial composition and expenditure of energy during food-based competition. Nevertheless, the apparent effects of SD (stocking density) on growth, survival rate, and productivity among the treatments provided by BFT were insignificant (*p* > 0.05), suggesting that it would be possible to use BFT to cultivate *O. pabda* in sub-tropical and/or tropical environments.

### 4.3. Proximate Composition

Biofloc has a wide variety of beneficial nutrients [68]; a significant amount of protein, fat, fiber, and ash can be found in biofloc [69]. The nutritional parameters of the biofloc in this investigation were comparable to those reported in earlier studies: 30.4% crude protein [69] and 2.11–5.4% lipid [23,68,69]. The crude protein value of the examined floc was in the range of 27.8–58% as was reported in another study conducted with BFT [68].

According to the results of this investigation, the biofloc groups had a significantly increased crude protein and lipid content of *O. pabda* carcasses compared to the traditional groups, which is similar to the findings reported by [70,71,72]. Biofloc has been shown to be nutritionally perfect for fish growth in previous studies [73]. Zhang et al. [73] also implicated that microbial biofloc leads to dietary protein savings without altering growth characteristics or biochemical profiles of fish carcasses. Previous studies also illustrated that different carbon sources in biofloc systems not only could influence the biochemical composition of bioflocs, but also could influence the proximate composition of the cultured species [74,75,76]. Studies have also evident that species like *Lepomis macrochirus* [77], *Cyprinus carpio* [71], *Clarias gariepinus* [78], *Labeo rohita* [79], and *Oreochromis niloticus* [80,81] cultured in BFT system showed better biochemical composition due to the consumption of biofloc as an extra nutrition source. In this study, the biofloc produced in the BFT tanks might have contributed to the nutrition of fishes reared in the BFT tanks; accordingly, bioflocs can be a great source of natural food containing high protein and lipids for cultivating pabda fishes in the tanks.

The percentages of EAAs and TAAs in the fish samples in this investigation were higher for the BFT treatments than for the traditional system. Sontakke et al. [82] reported that milkfish reared in biofloc had a greater EAAs and TAAs than milkfish reared in clear water. The findings are additionally corroborated by the investigation conducted by Ai et al. [83] and Hu et al. [84] on the development of large yellow croaker *Pseudosciaena crocea*, and black carp *Mylopharyngodon piceus*. In relation to specific AAs, The BFT-reared *O. pabda* exhibited higher concentrations of valine, cysteine, and proline, which may potentially contribute to enhanced growth as well as health, as suggested by Zhang et al. [85].

In terms of fatty acid composition, the *O. pabda* fishes reared in the BFT tanks were abundant in fatty acids; particularly, saturated fatty acids (e.g., palmitic acid and stearic acid), and n − 3 and n − 6 fatty acids (e.g., α-linolenic acid, arachidonic acid, and docosahexaenoic acid) were found in rich quantities in BFT-cultured fishes. However, the docosahexaenoic acid level recorded in the BFT-reared fishes was much greater compared to the 6.43–8.01% reported by Sontakke et al. [82] and 2.5% by Ridha et al. [86]. In comparison with the fish of the TS tanks, a higher content of, α-linolenic acid, arachidonic acid, and docosahexaenoic acid in the BFT-reared *O. pabda* fish might be linked to the biofloc composition. This study has also deduced that the BFT treatment changed the fatty acid composition of *O. pabda* flesh by raising the content of n − 3 and n − 6 polyunsaturated fatty acids, which helped the fish grow faster, survive longer, and be less stressed, as suggested by Li et al. [87] and Zahid et al. [88]. It can also be mentioned that Sontakke et al. [82] discovered that BFT generates higher levels of EAAs and total amino acids, which indicated that different aquatic bacteria utilize the nitrogen and other resources in the water for biosynthesis, resulting in distinct protein compositions and amino acid (AA) contents [89,90,91].

### 4.4. Hematological Parameter

Hematological indicators are evaluated along with growth performance to measure the physiological state of aquatic animals under specific dietary and environmental conditions as well as the general health of fish [92]. The physiological characteristics of fish are influenced by various aspects, including their ambient temperature [93] and overall health state [94]. In the current investigation, the higher levels of RBCs, Hb, and hematocrit but lower level of glucose in the BFT-reared fishes indicate a positive effect of BFT on the physiological conditions as addressed by Debbarma et al. [43]. Fish blood chemistry changes are likely related to various environmental factors, including type or quantity of organic carbon sources, type and quality of floc, and quality of bioactive compounds in biofloc [95]. The current study highlighted the positive influence of bioflocs on immunity of fish species as documented in Ahmad et al. [79]. Similarly, Haghparast et al. [96] reported enhanced hematological performance in the biofloc treatment groups for *Cyprinus carpio*.

### 4.5. Economic Evaluation

Production economics describes the trade-off between a fish cultivation operation’s economic revenue and expenditures. Although the increase in stocking density resulted in greater expenses for feed, fingerlings, and other related costs, both the rearing systems exhibited a somewhat higher benefit–cost ratio (BCR) when a stocking density of 22 fry/m^2^ was employed. It is of note that the lowest FCR was likely the driving factor, making the highest BCR for the BFT system with a stocking density of 22 fry/m^2^. These results were supported by other studies claiming the BFT system as more practical and effective for commercial fish farming [97,98]. Sontakke and Haridas [97] found that the 30-day nursery phase of milkfish fry grown to fingerling size in the biofloc-based system was economically viable with the higher net income. The feeding cost from the biofloc system was comparatively less than the traditional culture since biofloc has adequate protein, lipid, carbohydrate, and ash contents, which could be used as a fish feed [98].

## 5. Conclusions

The use of biofloc technology in aquaculture is relatively new, but it has already shown promising results in waste recycling, microbial protein supplementation, and yield enhancement of fish and shellfish farms. This research demonstrated that the biofloc system improved the overall quality of the water, as well as survivability, nutritional contents, growth, and economic return of the *O. pabda*. This study concludes that a density of 22 fry/m^2^ is optimal for cultivating *O. pabda* fish in a BFT system under the specified environmental conditions, based on productivity, feed conversion ratio, and profitability indices. On a global scale, the current study suggests that fish farmers may use this stocking density for increasing their pabda production per unit area; in total, 2.1–2.3 tonne pabda/ha can be produced in this BFT system while 1.5–2.0 tonne/ha production has been recorded in the farming system being currently used by the farmers. The significant data of this study can be effectively used in advanced studies of *O. pabda* fish as well as other catfish species culture intensification with special reference to artificial feed development, nutritional enhancement, pathogen control, and biosecurity implementation. In addition, non-significant production statistics could be considered in further technological advancement in aquaculture. However, the findings call for additional research to identify the best source(s) of carbohydrates and the ideal protein content of feed used in an *O. pabda* BFT system.

## Figures and Tables

**Table 1:** Physico-chemical parameters of water in the biofloc technology and the traditional rearing tanks with different stocking densities (Mean ± SD).

Parameters	Traditional		Biofloc		
	TS_1_	TS_2_	BFTS_1_	BFTS_2_	BFTS_3_
Temperature (°C)	26.27 ± 0.13 ^a^ (18.6–29.43)	26.25 ± 0.13 ^a^ (18.17–29.57)	26.23 ± 0.12 ^a^ (18.47–29.47)	26.21 ± 0.12 ^a^ (18.63–29.43)	26.22 ± 0.13 ^a^ (18.57–29.43)
DO (mg L^−1^)	6.6 ± 0.11 ^a^ (5.67–7.43)	6.57 ± 0.09 ^a^ (5.57–7.47)	5.93 ± 0.11 ^b^ (4.87–7.70)	5.89 ± 0.12 ^b^ (4.77–7.53)	5.78 ± 0.13 ^b^ (4.70–7.37)
TAN (mg L^−1^)	0.31 ± 0.03 ^a^ (0.00–0.71)	0.32 ± 0.03 ^a^ (0.00–0.71)	0.25 ± 0.02 ^b^ (0.00–0.63)	0.27 ± 0.02 ^b^ (0.00–0.62)	0.27 ± 0.02 ^b^ (0.00–0.63)
pH	7.95 ± 0.1 ^a^ (7.23–8.57)	7.95 ± 0.1 ^a^ (7.2–8.57)	7.63 ± 0.11 ^b^ (7.00–8.53)	7.61 ± 0.1 ^b^ (7.00–8.54)	7.71 ± 0.09 ^b^ (7.00–8.50)
TDS (mg L^−1^)	347.17 ± 5.75 ^a^ (290–401.67)	352.31 ± 4.78 ^a^ (286.67–406.67)	1203.28 ± 6.17 ^b^ (270–1346.67)	1190.03 ± 0.17 ^c^ (270–1348.33)	1211.57 ± 7.12 ^b^ (270–1346.67)
NO_2_ (mg L^−1^)	0.29 ± 0.02 ^a^ (0.00–0.50)	0.31 ± 0.02 ^a^ (0.00–0.52)	0.19 ± 0.03 ^b^ (0.00–0.41)	0.18 ± 0.02 ^b^ (0.00–0.43)	0.18 ± 0.02 ^b^ (0.00–0.43)
NO_3_ (mg L^−1^)	1.66 ± 0.2 ^a^ (0.00–2.77)	1.99 ± 0.21 ^a^ (0.00–3.40)	0.88 ± 0.11 ^b^ (0.00–1.68)	0.86 ± 0.12 ^b^ (0.00–1.67)	1.09 ± 0.12 ^c^ (0.00–2.08)
Alkalinity (mg L^−1^)	137.35 ± 3.44 ^a^ (110.0–162.67)	151.87 ± 4.11 ^a^ (110.0–179.33)	164.19 ± 3.79 ^b^ (110.0–191.67)	171.5 ± 3.09 ^b^ (110.0–194.67)	169.78 ± 4.39 ^b^ (110.0–197.33)
Hardness (mg L^−1^)	137.35 ± 3.99 ^a^ (110.67–189.67)	136.7 ± 3.61 ^a^ (111.33–173.33)	143.56 ± 5.27 ^b^ (112.67–175.33)	130.04 ± 4.35 ^c^ (105.33–174.67)	127.06 ± 4.12 ^c^ (107.33–170.00)
Salinity (ppt)	0.3 ± 0 ^a^ (0.30–0.30)	0.3 ± 0 ^a^ (0.30–0.30)	1.15 ± 0 ^b^ (0.30–1.20)	1.06 ± 0 ^b^ (0.30–1.10)	1.15 ± 0 ^b^ (0.30–1.20)
Floc Volume (ml L^−1^)	-	-	14.57 ± 0.2 ^a^ (0.00–26.20)	14.15 ± 0.3 ^a^ (0.00–26.00)	13.97 ± 0.35 ^a^ (0.00–26.47)
BOD (mg L^−1^)	2.01 ± 0.22 ^a^ (1.67–2.33)	2.07 ± 0.17 ^a^ (1.83–2.27)	3.79 ± 0.23 ^b^ (2.10–4.23)	3.68 ± 0.19 ^b^ (2.37–4.17)	3.8 ± 0.17 ^b^ (2.43–4.37)

Data in the same row bearing different superscript letters indicate a significant difference (*p* < 0.05). DO—dissolved oxygen, TAN—total ammonia nitrogen, TDS—total dissolved solids, NO_2_—nitrite, NO_3_—nitrate, BOD—biological oxygen demand.

**Table 2:** Comparison of fatty acid profile % of *O. pabda* from the biofloc (BFT) and the traditional system (TS) rearing tanks (Range).

Fatty Acids (Area %)	Culture System	
	Biofloc	Traditional
SFA		
C16: 0 Palmictic acid	24.22–25.98 ^a^	7.99–8.12 ^b^
C14: 0 Myristic acid	0.68–0.75	ND
C18: 0 Stearic acid	4.87–6.04 ^a^	0.50–0.58 ^b^
C20: 0 Arachidic acid	7.09–7.79 ^a^	27.7–29.09 ^b^
C23: 0 Tricosanoic acid	0.69–0.73 ^a^	0.79–0.84 ^b^
∑SFA=	38.78–40.2 ^a^	37.23–38.48 ^b^
MUFA		
C14: 1 Myristoleicacid	2.5–2.52 ^a^	2.4–2.55 ^a^
C18: 1 Oleic acid	7.12–8.09 ^a^	8.97–10.75 ^b^
C20: 1 Eicosenoic acid	5.2–6.18 ^a^	7.91–8.2 ^b^
C22: 1 Erucicacid	7.67–8.02 ^a^	7.91–8.2 ^a^
∑ MUFA=	27.34–29.61 ^a^	22.85–24.05 ^b^
PUFA		
C18: 2 Linoleic acid	4.04–5.16 ^a^	4.88–5.39 ^a^
C18: 3 γ-Linolenicacid	1.18–2.34 ^a^	2.19–2.24 ^a^
C18: 3 α-Linolenic acid	3.49–3.56 ^a^	7.99–8.12 ^b^
C20:3 Eicosatrienoic acid	0.58–0.60 ^a^	1.11–1.18 ^b^
C20: 2 cis-11, 14 Eicosadienoic acid	0.57–0.59 ^a^	1.07–1.12 ^b^
C20: 4 Arachidonic acid	7.09–7.79 ^a^	8.97–10.75 ^b^
C20: 5 Eicosapentaenoic (EPA)	1.68–1.72 ^a^	0.79–0.84 ^b^
C22: 6 Docosahexaenoic (DHA)	7.85–7.93 ^a^	7.99–8.12 ^b^
∑PUFA=	35.05–37.67 ^a^	27.45–28.83 ^b^
n − 3/n − 6	1.07–1.11 ^a^	1.13–1.17 ^b^

Data in the same row bearing different superscript letters indicate a significant difference (*p* < 0.05). SFA: saturated fatty acid, MUFA: monounsaturated fatty acid, PUFA: polyunsaturated fatty acid.

**Table 3:** Amino acid composition (%) of *O. pabda* reared in biofloc (BFT) and traditional system (TS) tanks (Range).

Amino Acids (%)	Rearing System	
	Biofloc	Traditional
EAA (%)		
Threonine	5.79–6.18 ^a^	5.58–5.97 ^a^
Valine	15.98–16.14 ^a^	5.60–5.70 ^b^
Arginine	ND	2.78–2.94
Histidine	26.13–28.04 ^a^	3.55–4.20 ^b^
Lysine	5.13–5.18 ^a^	14.25–14.60 ^b^
Phenylalanine	4.52–4.66 ^a^	6.94–7.12 ^b^
Methionine	7.43–7.77 ^a^	3.31–3.60 ^b^
Isoleucine	3.04–3.25 ^a^	4.18–4.90 ^b^
Leucine	5.06–5.10 ^a^	11.87–13.53 ^b^
∑EAA=	73.36–75.44 ^a^	59.47–60.70 ^b^
NEAA (%)		
Glycine	ND	8.58–9.14
Glutamic acid	17.22–17.90 ^a^	18.05–18.38 ^b^
Alanine	0.02–0.04 ^a^	11.08–12.77 ^b^
Aspartic acid	13.78–15.12 ^a^	14.61–15.15 ^a^
Serine	3.34–3.58 ^a^	3.12–3.33 ^a^
Tyrosine	18.26–18.38 ^a^	4.80–5.04 ^b^
∑NEAA=	52.75–54.90 ^a^	61.10–62.92 ^b^
CEAA (%)		
Cysteine	14.13–15.04 ^a^	0.01–0.03 ^b^
Proline	2.88–3.10 ^a^	7.48–7.50 ^a^
∑CEAA=	17.21–17.92 ^a^	7.50–7.52 ^b^

Data in the same row bearing different superscript letters indicate a significant difference (*p* < 0.05). EAA: essential amino acid, NEAA: non-essential amino acid, CEAA: conditionally essential amino acid.

**Table 4:** Parameters of *O. pabda* growth and production in the biofloc (BFT) and the traditional rearing system (TS) for the culture period (90 days). (Mean ± SD).

Variables	Culture System					Two-Way Anova (Corrected Model: Type III)		
	TS_1_	TS_2_	BFTS_1_	BFTS_2_	BFTS_3_	CS	SD	CS × SD
IBW (g)	0.30 ± 0.02 ^a^	0.31 ± 0.03 ^a^	0.29 ± 0.01 ^a^	0.30 ± 0.02 ^a^	0.31 ± 0.03 ^a^	ns	*	ns
FBW (g)	7.99 ± 0.17 ^b^	7.15 ± 0.58 ^b^	12.31 ± 0.93 ^a^	10.04 ± 0.14 ^c^	9.98 ± 0.94 ^c^	*	*	ns
IL (cm)	3.40 ± 0.37 ^a^	3.37 ± 0.15 ^a^	3.33 ± 0.29 ^a^	3.43 ± 0.21 ^a^	3.33 ± 0.15 ^a^	ns	ns	ns
FL (cm)	11.57 ± 0.21 ^c^	11.17 ± 0.25 ^c^	13.10 ± 0.30 ^a^	12.50 ± 0.20 ^b^	12.37 ± 0.46 ^b^	*	*	ns
WG (g)	7.69 ± 0.19 ^c^	6.85 ^d^ ± 0.57 ^d^	12.02 ± 0.93 ^a^	9.73 ± 0.13 ^b^	9.67 ± 0.96 ^b^	*	*	ns
SGR (%/day)	3.64 ± 0.22 ^c^	3.51 ± 0.05 ^c^	4.11 ± 0.17 ^a^	3.89 ± 0.03 ^a^	3.88 ± 0.3 ^a^	*	*	ns
FCR	1.68 ± 0.06 ^a^	1.73 ± 0.12 ^a^	1.31 ± 0.06 ^c^	1.27 ± 0.04 ^c^	1.51 ± 0.15 ^b^	*	*	ns
SR (%)	90.83 ± 3.82 ^ac^	86.67 ± 5.03 ^bc^	98.33 ± 2.89 ^a^	96.67 ± 3.06 ^a^	87.22 ± 4.20 ^ab^	*	*	ns
Initial biomass (kg/m^2^)	0.005 ± 0.34 ^a^	0.005 ± 0.43 ^a^	0.005 ± 0.20 ^a^	0.005 ± 0.35 ^a^	0.005 ± 0.49 ^a^	ns	ns	ns
Final biomass (kg/m^2^)	0.127 ± 0.01 ^c^	0.137 ± 0.10 ^c^	0.214 ± 0.02 ^ab^	0.215 ± 0.01 ^ab^	0.231 ± 0.02 ^a^	*	ns	ns
Biomass gain (kg/m^2^)	0.122 ± 0.01 ^c^	0.132 ± 0.01 ^c^	0.209 ± 0.01 ^ab^	0.210 ± 0.01 ^ab^	0.226 ± 0.02 ^a^	*	ns	ns

* Data in the same row bearing different superscript letters indicate a significant difference (*p* < 0.05). IBW—initial body weight, FBW—final body weight, IL—initial length, FL—final length, WG—weight gain, FCR—feed conversion ratio, SGR—specific growth rate, SR—survival rate, ns—non-significant.

**Table 5:** Hematological parameters of *O. pabda* collected from the biofloc (BFT) and the traditional culture system (TS) tanks (mean and range).

Blood Parameter	Biofloc	Traditional
RBC (×10^6^)/mm^3^	3.28 ± 0.28 ^a^ (2.98–3.52)	2.49 ± 0.19 ^b^ (2.3–2.68)
WBC (×10^3^)/mm^3^	43.49 ± 0.82 ^a^ (42.54–43.98)	42.38 ± 0.47 ^a^ (41.96–42.89)
Hb (g/dL)	5.04 ± 0.26 ^a^ (4.87–5.34)	2.66 ± 0.69 ^b^ (1.87–3.14)
Hematocrit (%)	34.5 ± 0.79 ^a^ (33.89–35.39)	23.7 ± 1.62 ^b^ (21.88–24.98)
Glucose (mmol/L)	9.06 ± 1.32 ^b^ (7.6–10.16)	15.89 ± 1.72 ^a^ (14.09–17.51)
Cholesterol (mg/dL)	114.98 ± 5.36 ^a^ (110.56–120.94)	109.74 ± 2.22 ^a^ (107.45–111.59)

Data in the same row bearing different superscript letters indicate a significant difference (*p* < 0.05). RBC—red blood cell, WBC—white blood cell, Hb—hemoglobin.

**Table 6:** Benefit–cost analysis of *O. pabda* from the biofloc culture (BFT) and traditional culture system (TS) with different stocking densities (in USD, FOREX 1$ = 105.65 BDT; Dated: 15.03.2023).

Operating Items	Life Period (Years) (2 Cycle/Year)	Traditional		Biofloc		
		TS_1_	TS_2_	BFTS_1_	BFTS_2_	BFTS_3_
Aerator pump (0.08 hp)	5	0.14	0.14	0.14	0.14	0.14
Air stone	3	0.04	0.04	0.04	0.04	0.04
Silicon pipe	2	0.04	0.04	0.04	0.04	0.04
Net	2	0.02	0.02	0.02	0.02	0.02
Weighing scale	5	0.04	0.04	0.04	0.04	0.04
Others	2	0.05	0.05	0.05	0.05	0.05
Total fixed cost		0.33 ^a^	0.33 ^a^	0.33 ^a^	0.33 ^a^	0.33 ^a^
Fry	-	0.38	0.47	0.38	0.47	0.57
Feed	-	0.33	0.37	0.47	0.49	0.58
Molasses	-	-	-	0.091	0.094	0.113
Lime	-	-	-	0.001	0.001	0.001
Salt	-	-	-	0.11	0.11	0.11
Total variable cost		0.71 ^c^	0.84 ^c^	1.05 ^b^	1.17 ^ab^	1.38 ^a^
Total cost		1.04 ^c^	1.17 ^c^	1.39 ^b^	1.50 ^ab^	1.71 ^a^
Total revenue		1.03 ± 0.04 ^d^	1.23 ± 0.07 ^c^	1.58 ± 0.05 ^b^	1.83 ± 0.05 ^a^	1.98 ± 0.09 ^a^
BCR		0.99 ± 0.04 ^c^	1.05 ± 0.06 ^c^	1.14 ± 0.03 ^b^	1.22 ± 0.04 ^a^	1.16 ± 0.05 ^a^

Data in the same row bearing different superscript letters indicate a significant difference (*p* < 0.05). BCR—benefit–cost ratio.

## Data Availability

Data are contained within the article.
